# First off Label Endovascular Clinical Experience to Treat Diffuse Cerebral Venous Sinus Thrombosis Using the INARI FlowTriever System: Case Report

**DOI:** 10.3389/fneur.2021.778842

**Published:** 2021-12-17

**Authors:** Saif Bushnaq, Samer Abdul Kareem, Nicholas Liaw, Bader Alenzi, Muhammad Khaleeq Ahmed, Merna Bushnaq, Osama O. Zaidat

**Affiliations:** ^1^Texas Tech University Health Sciences Center, Lubbock, TX, United States; ^2^St Vincent Mercy Hospital, Neuroscience Institute, Toledo, OH, United States; ^3^Medical College of Georgia, Augusta, GA, United States; ^4^King Hussein Cancer Center, Amman, Jordan

**Keywords:** cerebral venous sinus thrombosis, INARI FlowTriever system, endovascular treatment, intervention, case report

## Abstract

Anticoagulation with heparin is the current mainstay treatment for Cerebral Venous Sinus Thrombosis (CVST). Endovascular treatment is increasingly being used to treat patients with CVST who are non-responsive to anticoagulation. These more aggressive interventions include catheter-based local chemical thrombolysis, balloon angioplasty and mechanical thrombectomy with uncertain safety and efficacy. Here we describe the first reported clinical experience using the INARI FlowTriever system to treat a patient presented with focal weakness and found to have diffuse CVST.

## Introduction

Cerebral Venous Sinus Thrombosis (CVST) is a rare stroke with a wide range of symptomatology at presentation ranging from headache, focal weakness, and coma. Anticoagulation remains the mainstay of treatment. However, in a subset of patients; endovascular treatment can be potentially beneficial. Here we describe the first reported clinical experience using the INARI FlowTriever system to treat a patient presented with focal weakness and found to have diffuse CVST.

## Case Report

A 78-year-old female with past medical history including autoimmune hepatitis, hypothyroidism. She presented to the hospital via emergency medical services with left arm weakness and jerky movements. This event was witnessed by family while she was eating. No recent trauma or fall. No earache, hearing loss, or discharge. No loss of consciousness reported. Of note, she is on azathioprine for autoimmune hepatitis. She was evaluated by the stroke team upon arrival. Vital signs included: elevated blood pressure at 153/72 mmHg, normal pulse 91, and normal respiratory rate at 17. She was afebrile. Laboratory work up revealed normal white cell count (WBC) of 7.2 10^9^/L, and normal hemoglobin of 12 gm/dL. Platelets noted to be low at 80 10^9^/L. Serum chemistry was unremarkable except for low sodium of 129 mEq/L. Urine toxicology drug screen was negative. COVID-19 PCR (polymerase chain reaction) test was negative.

**Figure 1:**
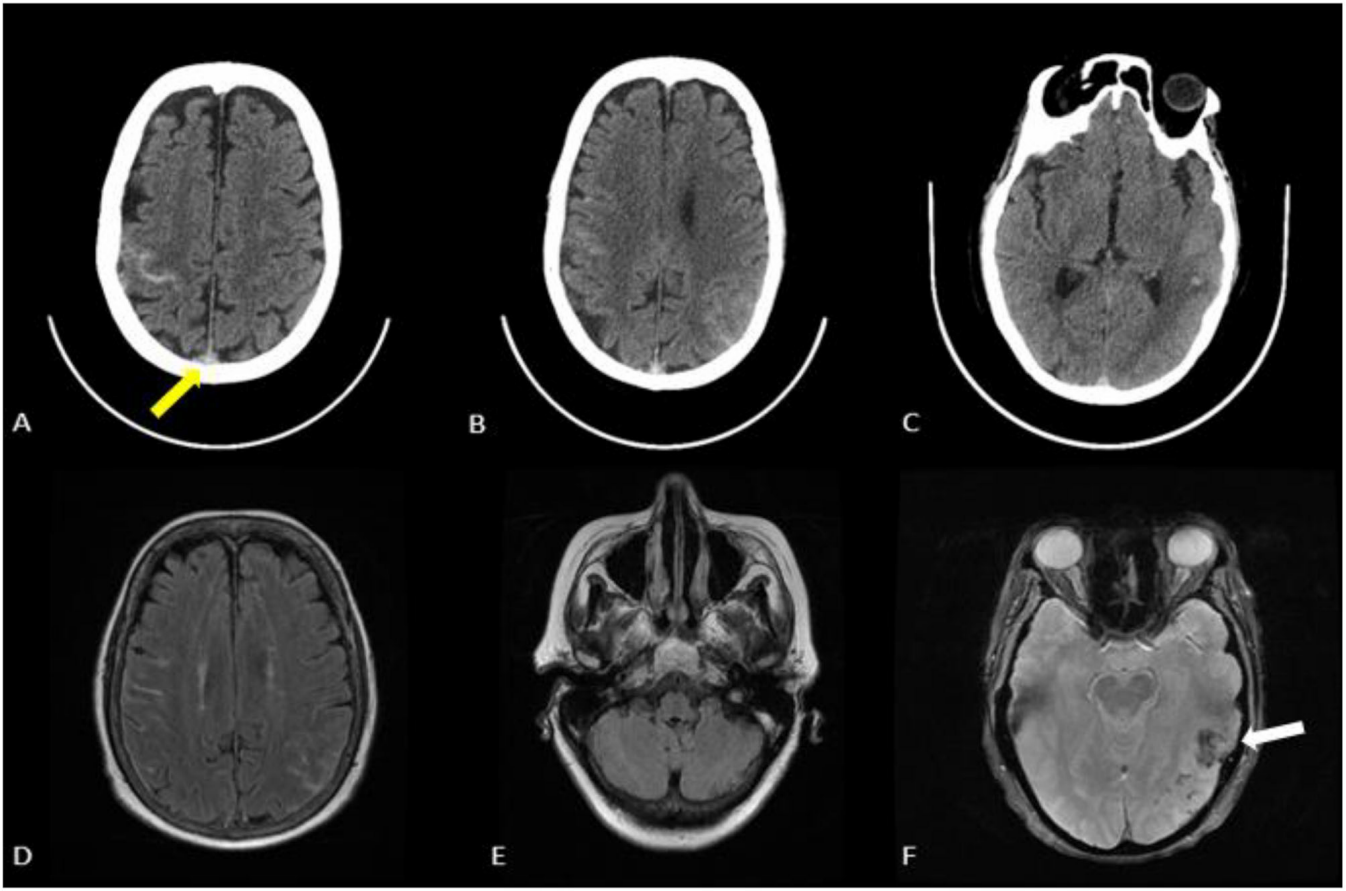
**(A–C)** CT head revealing acute multicompartment hemorrhages with left temporal parietal intraparenchymal hemorrhage measuring approximately 3 cm, right frontal subarachnoid hemorrhage and left parietal subarachnoid hemorrhage. No midline shift noted. Hyperdense sign noted (yellow arrow) in the superior sagittal sinus suggesting venous sinus thrombosis. **(D,E)** MRI brain without contrast FLAIR sequence with right frontal hyperintensity and left parietal hyperintensity consistent with subarachnoid hemorrhage. **(F)** Left temporal blooming noted (white arrow) on the GRE sequence consisted with intraparenchymal hemorrhage.

A Computed Tomography (CT) head on admission revealed left temporoparietal intraparenchymal hemorrhage, right frontal sulcal subarachnoid hemorrhage, and left parietal sulcal subarachnoid hemorrhage. Vessel images with Computed Tomography Angiogram (CTA) head and neck revealed extensive venous sinus thrombosis involving superior sagittal sinus, bilateral transverse, and sigmoid sinuses. She subsequently underwent Magnetic Resonance Imaging (MRI) of the brain with and without contrast and Magnetic Resonance Venogram (MRV) which confirmed extensive venous sinus thrombosis and multicompartment bleeding. No restricted diffusion noted (Figure 1).

Patient was evaluated by interventional neurology, neurosurgery, and neuro critical care team. She was started on levetiracetam for symptomatic treatment of focal seizures with left upper extremity shaking. She was started on anticoagulation with heparin drip and was admitted to neuro ICU for close neurological monitoring.

Desired therapeutic level of activated Partial Thromboplastin Time (aPTT) at 67.2 s was achieved at 24 h and patient remained in the neuro critical intensive care unit. After a thorough multidisciplinary team discussion due to persistent left-sided weakness, diffuse CVST, multicompartment bleeding while being on anticoagulation, low platelets, and anticipation of moderate to high risk of unfavorable outcome; the decision was to perform endovascular mechanical venous thrombectomy (Approximately 48 h after admission). She underwent a successful mechanical venous thrombectomy using the INARI FlowTriever system with large clot burden extracted. She remained clinically stable after the procedure and her left upper extremity weakness improved at day 5. No new symptomatic ICH. The 22 French (7.33 mm) venous access was sutured with figure of 8 technique followed by manual pressure. No post-procedure groin complications noted. She was switched to novel oral anticoagulation prior to discharge. During the 3 months follow-up–MRI brain with and without contrast revealed near complete resolution of the clot burden in superior sagittal sinus and left transverse-sigmoid complex. Her 3 months modified Rankin score was at 0. She was resumed on apixaban for 12 months with a follow-up brain magnetic resonance venogram planned.

## Mechanical Endovascular Venous Thrombectomy: Procedure Details

The patient was brought to the interventional suite by the anesthesia team and placed in a supine position on the angiogram table. Patient arrived intubated. The right radial region was prepped, draped and cleaned in a sterile fashion. The right radial artery was accessed with ultrasound guidance using a micropuncture needle and a 5 French by 10 cm introducer sheath was placed. Radial cocktail was given to minimize vasospasm. Subsequently, a 5 French glide catheter was advanced over a Glidewire to select the right internal carotid artery under fluoroscopy guidance. Right internal carotid angiogram run revealed filling defects in the mid to posterior aspect of superior sagittal sinus, right transverse, and right sigmoid sinus consistent with diffuse venous sinus thrombosis. There is venous congestion and no venous outflow noted in the left transverse and sigmoid sinuses.

**Figure 2:**
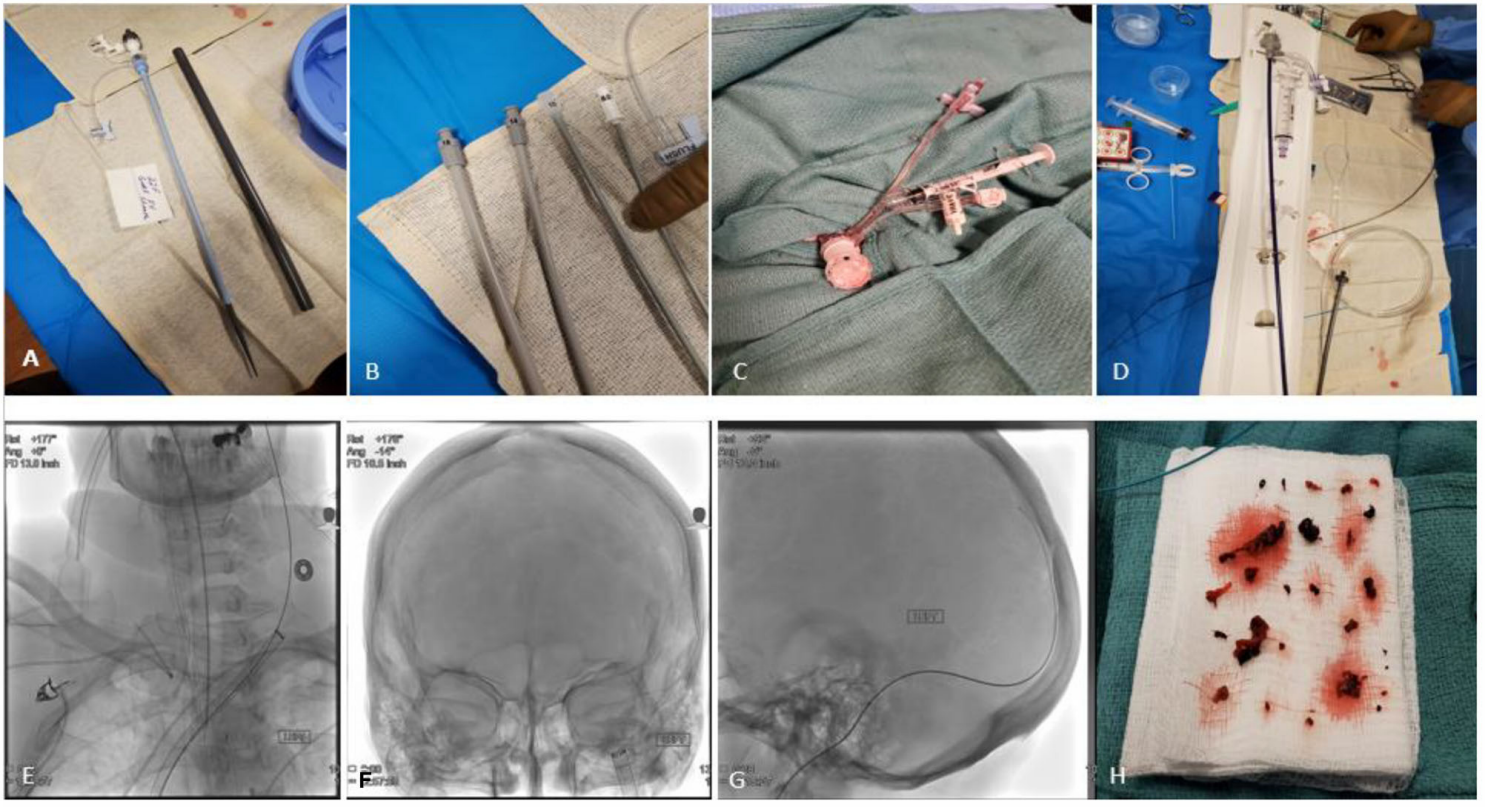
Transvenous endovascular mechanical thrombectomy using the INARI FlowTriever system. **(A–C)** Right femoral venous access was obtained by placing a 22F GORE DrySeal Flex Introducer Sheath with pre-dilation using 8F, 10F, 14F then 18F dilators. 2 cc of air was inflated in the GORE DrySeal Flex valve to stop back flow of venous blood. **(D,E)** After selecting the left internal jugular vein; The INARI Triver20 large bore aspiration catheter was advanced with its dilator over the TAD2 exchange wire (Over-the-wire technique) and then the dilator was removed. **(F–H)** large volume venous clots retrieved using the Vect074 reperfusion catheter and Solitaire stent retriever and negative pressure applied with a 60 cc syringe through the INARI Triver20 large bore aspiration catheter. Anticoagulation with heparin was resumed after the procedure.

The right common femoral vein was accessed with ultrasound guidance using a single wall needle technique and a 5 French short sheath was placed. Subsequently an 8 French, 10 French, 14 French and then 18 French dilators were sequentially used before introducing a 22 French venous sheath (GORE DrySeal Flex Introducer Sheath). The sheath was connected to a syringe and inflated to stop venous blood backflow. Then a 5 French select catheter was advanced over a Glidewire to the right atrium, superior vena cava, then to select the left internal jugular vein. The Glidewire was subsequently removed and TAD2 tapered peripheral wire (0.035–0.018 260 cm) was advanced through the select catheter and parked in the distal left internal jugular vein at close proximity to the jugular bulb level. Then the INARI Triver20 was advanced over the TAD2 (Over-the-wire technique) to the left jugular bulb. Of note, there was no difficulty noted to advance the large bore aspiration catheter (INARI Triver20) into the internal jugular vein over the stiff TAD2 wire. Then under fluoroscopic guidance the Rebar 021 microcatheter was advanced over Victory 18 microguidewire into the superior sagittal sinus crossing the thrombus. Then solitaire stent retriever 6 x 40 mm was advanced through the Rebar microcatheter and was successfully deployed across the mid aspect of the superior sagittal sinus. The Rebar microcatheter was then removed. Multiple attempts to advance the FlowTriver Catheter over the solitaire wire through the INARI Triver20 into the left transverse sigmoid complex was unsuccessful. Amplatz Super Stiff wire 0.035 × 260 cm was then used to advance the INARI Triver20 aspiration catheter further distally. However, it was challenging to cross the jugular foramen with either the INARI Triver20 or the FlowTriver Catheter. Subsequently the FlowTriver Catheter was removed and Vect074 reperfusion catheter was advanced over the Solitaire stent retriever wire through the Triver20 large bore aspiration catheter, then the Vect074 was advanced to the proximal aspect of the Solitaire stent retriever in the mid posterior sagittal sinus (was advanced back and forth over the Solitaire stent retriever wire under continuous aspiration with negative pressure connected to the penumbra pump through the Vect074). Then the Vect074 reperfusion catheter and Solitaire stent retriever was removed from the body as one piece through the Triver20 large bore aspiration catheter with negative pressure applied using a 60 cc syringe. Multiple large red clots noted in the the FlowTriver Catheter aspirate. Total fluoroscopy time is 50.8 min, and 70 cc Visipaque 270 low osmolar contrast was used (Figure 2: Procedure technique).

## Discussion

Here we discussed a patient with diffuse cerebral venous sinus thrombosis who was treated initially with large molecular weight heparin drip with a desired therapeutic aPTT level. The patient was deemed moderate to high risk of unfavorable outcome and was subsequently underwent mechanical endovascular venous thrombectomy using the INARI FlowTriever system and large clot burden was aspirated with a reasonable safety profile.

The FLARE (FlowTriever Pulmonary Embolectomy Clinical Study) is a prospective single-arm, multicenter investigational device trial in patients with acute intermediate risk pulmonary embolism using the INARI FlowTriever system (Inari Medical, Irvine, California) (1). This study revealed an acceptable effectiveness profile compared to catheter directed thrombolysis with an average right ventricle/left ventricular risk reduction of 0.38 (25% risk reduction), and a favorable safety profile with major adverse events rate at 3.8%. Major adverse events included any of the following within 48 h of treatment: device-related death, major bleeding, treatment-related clinical deterioration, treatment-related pulmonary vascular injury, and treatment-related cardiac injury.

Endovascular treatment for CVST remains a challenge in the neuro interventional field. Anticoagulation is the mainstay first line treatment for Cerebral Venous Sinus Thrombosis (CVS T) according to American stroke Association/American Heart Association and European guidelines (2, 3). However, a small subset of patients would potentially benefit from endovascular treatment but it still uncertain how to select these patients and what is the best timeline to offer early endovascular treatment (4, 5).

TO-ACT (Thrombolysis or Anticoagulation for Cerebral Venous Thrombosis) is the first multicenter randomized clinical trial in severe CVST (6). Patients with radiologically confirmed CVST who had at least 1 risk factor for a poor outcome (mental status disorder, coma state, intracerebral hemorrhage, or thrombosis of the deep venous system) were included. A total of 67 patients were randomized to endovascular venous thrombectomy (EVT) with standard medical care (intervention group) vs. standard medical care alone (control group). The study found in functional outcome at 12 months between both groups (RR ratio, 0.99; 95% CI, 0.71–1.38). However, TO-ACT is underpowered and should not be interpreted as definitive proof that EVT is ineffective in CVST. Our patient did meet TO-ACT inclusion criteria with presence of ICH.

The INARI medical FlowTriever system is the only FDA approved mechanical thrombectomy system indicated for the treatment of pulmonary embolism. It is specifically designed for venous clots. It is composed of a trackable large bore aspiration catheter; INARI Triver20 (available in other different sizes: Triver16: 16 French, Triver20: 20 French, and Triver24: 24 French), connected to a large volume syringe for manual aspiration designed to extract large volume of clots. The INARI FlowTriever Catheter; has 3 expanding nitinol mesh disks; available in 4 sizes: Small (6–10 mm), Medium (11–12 mm), (large 15–18 mm), and X large (19–25 mm), designed to engage and disrupt venous clots and subsequently deliver it to the large bore aspiration catheter. This FlowTriever size offers a larger diameter compared to other stentretrivers (Solitaire, Trevo) and might be more efficient to disrupt larger venous clots. However, this warrants further studies.

From a technical standpoint; It was feasible to advance the INARI FlowTriever system through the inferior vena cava, right atrium, superior vena cava, and into the left jugular bulb. However, we were unable to advance the Triever 20 aspiration catheter into the sigmoid sinus, even over the Vecta 071 while the solitaire device is deployed. The Triever 20 aspiration catheter and the flow triver is a bulky system. New changes has evolved in the Inari system. The Triever20 Curve has a customizable 260° bend designed for improved navigability and torqueability for challenging anatomy. The Triever24 comes now with FLEX technology to better navigate tortuous anatomies and smoothly deliver the catheter to the left pulmonary artery. These modifications might make it doable to track the Triever aspiration catheter distally into the sigmoid and transverse sinuses.

The 22 French (7.33 mm) venous access was sutured with figure of 8 technique. This was followed with manual pressure. No post-procedure groin complications noted. This illustrates that the common femoral vein can accommodate large catheters if needed (Common femoral vein approximate diameter is 8.2 ± 0.14 mm). In a multicenter, single-arm FLASH Registry evaluating real world patient outcomes after treatment of pulmonary embolism with FlowTriever; of the first 230 analyzed, there was a single case of access site complication (0.4%). There was 0% mortality throughout 48 h post-procedure, with 3 major bleeds (all non-ICH), and 0 intra-procedural device and procedure related adverse events (7).

**Figure 3:**
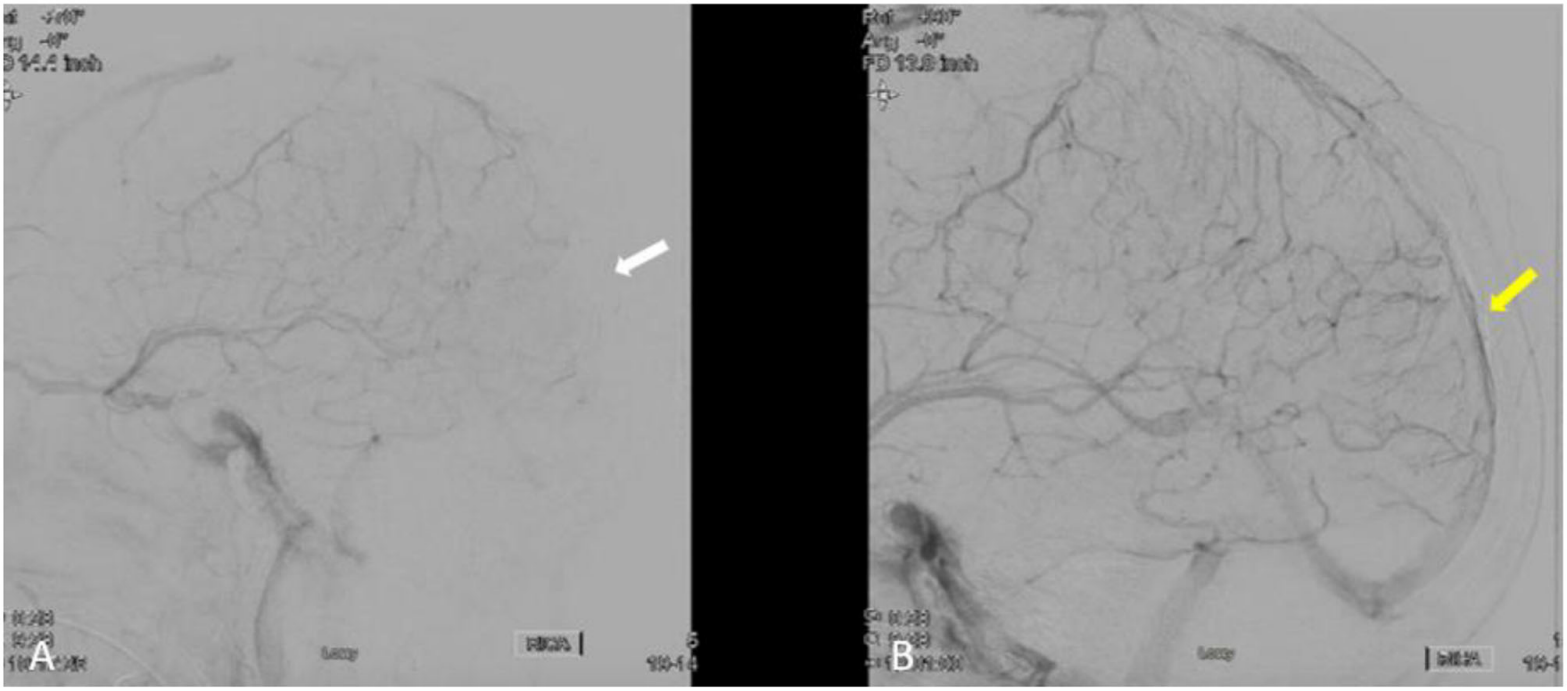
**(A,B)** Lateral cerebral angiogram run in **(B)** is post venous thrombectomy treatment demonstrating partial recanalization of posterior aspect of superior sagittal sinus and left transverse, and sigmoid sinuses (yellow arrow) compared to pre-treatment (white arrow in **A**).

**Figure 4:**
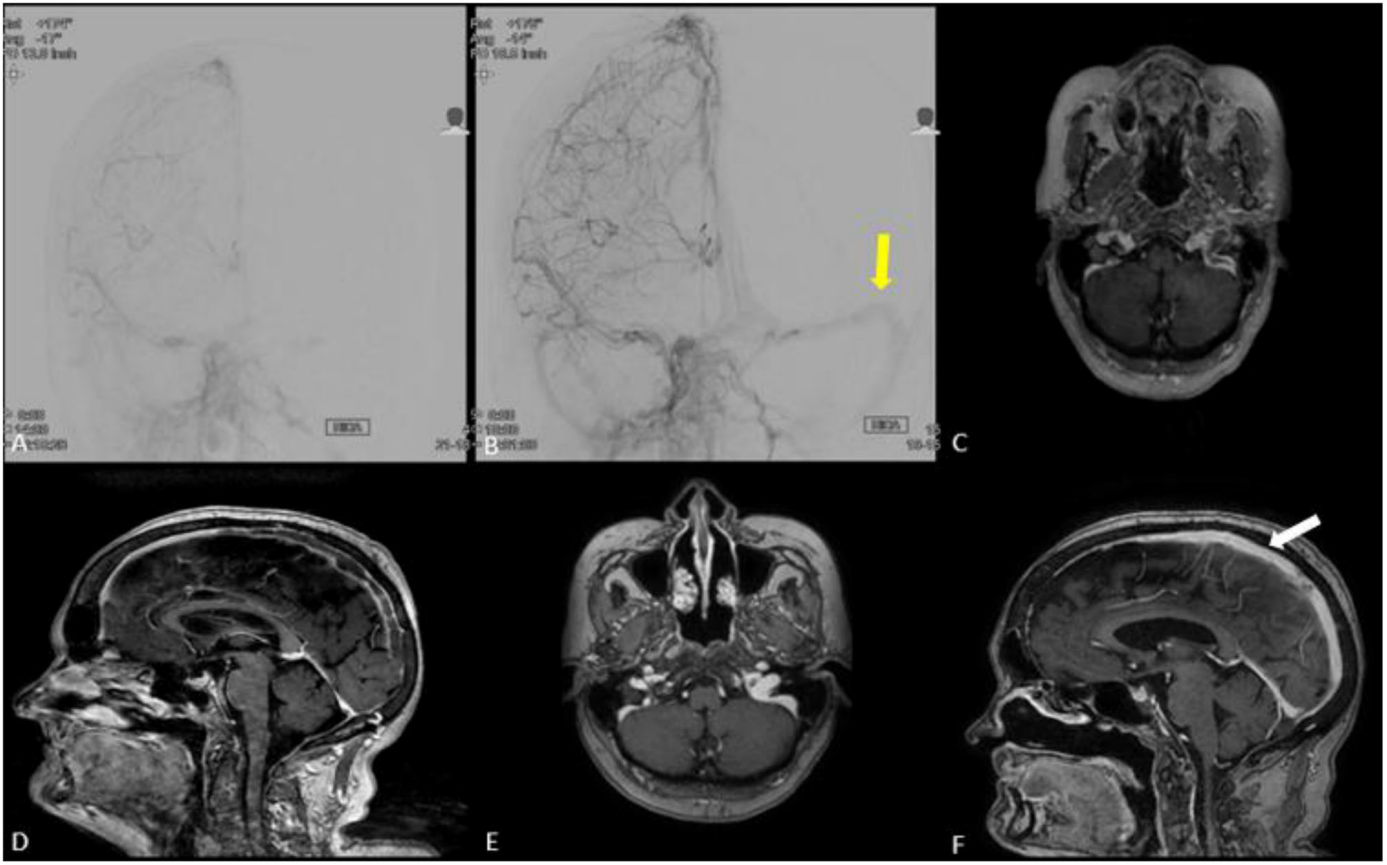
**(A,B)** Anteroposterior cerebral angiogram run in **(B)** is post treatment demonstrating partial recanalization of posterior aspect of superior sagittal sinus and left transverse/sigmoid complex (yellow arrow) compared to pre-treatment **(A)**. **(C–F)** MRI brain with and without contrast in axial **(E)** and coronal views **(F)** demonstrating near complete recanalization of superior sagittal sinus (white arrow) and left transverse/sigmoid complex at 3 months clinic follow-up compared to MRI brain obtained prior to venous thrombectomy **(C,D)**.

In our patient; the angiographic results revealed partial recanalization and improvement of venous flow after extracting a large clot burden. MRI brain at 3-months follow up demonstrated near complete recanalization of the venous sinuses (Figures 3, 4). Mechanical disruption of the venous clot in selected patients would probably decrease the intracranial pressure and give systemic medical anticoagulation (heparin drip, enoxaparin, novel oral anticoagulants) a better chance to stabilize and dissolve the clot.

Various neuro endovascular techniques has been attempted to treat cerebral venous sinus thrombosis. This includes direct aspiration thrombectomy, stent retriever thrombectomy, balloon thrombectomy, balloon angioplasty and stenting, AngioJet. However; it is unclear which approach and or devices provides the optimal restoration of venous blood flow; and further evidence is needed (8–10).

We believe that intracranial mechanical venous thrombectomy is still lacking behind compared endovascular thrombectomy for ischemic treatment. New innovations including catheters, stent retrievers, and procedure techniques is warranted to advance the field.

## Conclusion

Current neuro endovascular techniques and devices are not particularly designed for treatment of cerebral venous sinus thrombosis pathology and there is utmost need for further innovation and new devices. This case report illustrates a transvenous endovascular treatment of cerebral venous sinus thrombosis with an off-label use of the INARI FlowTriever System. There was no peri-procedure complications noted with a good clinical functional outcome. Further data are required from this population of interest with severe CVST.

## Data Availability Statement

The original contributions presented in the study are included in the article/supplementary material, further inquiries can be directed to the corresponding author.

## Ethics Statement

Written informed consent was obtained from the individual(s) for the publication of any potentially identifiable images or data included in this article.

## Author Contributions

SB, NL, and OZ contributed to conception and design of the study. SA contributed to data analysis. SB and MK wrote the first draft of the manuscript. All authors contributed to manuscript revision.

## Conflict of Interest

The authors declare that the research was conducted in the absence of any commercial or financial relationships that could be construed as a potential conflict of interest. The handling editor declared a past co-authorship with one of the authors OZ.

## Publisher's Note

All claims expressed in this article are solely those of the authors and do not necessarily represent those of their affiliated organizations, or those of the publisher, the editors and the reviewers. Any product that may be evaluated in this article, or claim that may be made by its manufacturer, is not guaranteed or endorsed by the publisher.
